# Assessment of In Vivo Analgesic, Anti-Inflammatory and Wound Healing Properties of Aqueous Leaf Extract of *Annona reticulata* Linn

**DOI:** 10.1155/tswj/4535663

**Published:** 2025-11-04

**Authors:** Tasnia Binte Bari Kabbo, Md. Sohel Rana, Pritesh Ranjan Dash

**Affiliations:** ^1^Department of Pharmacy, Jahangirnagar University, Dhaka, Bangladesh; ^2^Department of Pharmacy, Primeasia University, Dhaka, Bangladesh

**Keywords:** analgesic, *Annona reticulate*, anti-inflammatory, aqueous, leaf, wound healing

## Abstract

*Annona reticulata* Linn.'s aqueous leaf extract was studied for analgesic, anti-inflammatory and wound healing potentials in animal models. Comprehensive in vivo studies were conducted in a mouse model using three well-established methods for evaluating analgesic potential; in all three studies, the aqueous extract at 200 and 400 mg/kg body weight doses showed significant and promising effects. A 400 mg/kg dose of aqueous fraction, in the acetic acid–induced writhing test, demonstrated 75% inhibition of pain; in the hot plate test, exhibited 80% analgesic efficacy (90 min later of taking dose); and in the formalin-induced paw licking test, exhibited inhibition of pain responses values of 32.31% and 66.61% in acute and chronic phases, respectively. By performing the xylene-induced ear edema method and the cotton pellet–induced granuloma test, notable anti-inflammatory potential was also found in the test fraction. Two hundred and 400 mg/kg dosages of aqueous leaf fraction reduced ear edema and granuloma brought on by xylene and cotton pellets (*p* < 0.001). Remarkable wound healing activity was also noted in the test extract in the burn wound model; the observed epithelialisation period for 10% ointment of aqueous extract was 13 ± 0.32 days, whereas the epithelialisation period for standard medication silver sulfadiazine was 14.20 ± 0.38 days. Moreover, probable components responsible for achieving these potentials were identified by utilising GC-MS analytical data.

## 1. Introduction

Pain is characterised as an unpleasant emotional and sensory experience that is associated with or manifested in relation to tissue damage. Any member of the pharmacological class intended to reduce pain is referred to as an analgesic. These drugs have varying effects on the neurological and central peripheral systems [1]. Most analgesic drugs on the market today are synthetic, and negative side effects might arise from long-term usage of these medications [2, 3]. In order to produce more reasonably priced medications, efforts must be made to introduce new medicinal plants. Due to the discovery of several analgesic chemicals from various plant sources, scientists were able to identify this therapeutic region and find new molecules with improved pharmacokinetic and pharmacodynamic potentials [4]. In addition, the body's natural defence against tissue damage caused by chemicals, pathogens or physical stress is inflammation [5]. Nonsteroidal anti-inflammatory drugs are among the drugs that are prescribed most frequently to treat inflammatory ailments. In addition to their negative side effects, the two primary drawbacks of the potent anti-inflammatory drugs currently available on the market are their toxicity and the potential for symptom recurrence after quitting them. The development and assessment of medications for their ability to reduce inflammation are therefore crucial, and numerous efforts are being made to find anti-inflammatory drugs originated from medicinal plants [6]. Moreover, a wound occurs when the cellular, anatomical or functional continuity of live tissue is lost or disrupted. Wound healing, sometimes called wound repair, is the body's natural process of regenerating dermal and epidermal tissue [7]. The quest for healing agents has proven challenging, and a lot of study has been planned to produce more effective ones. Many medicinal herbs have been used to treat wounds in folk medicine [8–13]. Some of these plants have antibacterial and pro-wounding healing potentials; they are useful for wound treatment [14]. To control and treat wounds, plants or compounds derived from plants must be identified and formulated. Many herbal treatments have been used to treat and cure wounds over the years [15]. Furthermore, GC-MS analysis is frequently performed on plant extracts to identify and measure volatile and semivolatile chemical components. This approach gives a thorough chemical profile of the plant [16, 17]. One of the historically significant plants used to treat various ailments is *Annona reticulata* L. (Bullock's heart). It is a member of the *Annonaceae* family. From various portions of *Annona reticulata* L., several phytoconstituents have been discovered. The current study was conducted to assess aqueous leaf fraction from *Annona reticulata* L.'s analgesic, anti-inflammatory and wound healing potentials, while GC-MS analysis was also performed to identify phytochemicals behind these activities.

## 2. Materials and Methods

### 2.1. Preparation of Plant Extract

*Annona reticulata* L. leaves were gathered from Gokulnagar, Savar, Dhaka. The plant material (leaves) was sun-dried individually and then dried in a hot air oven (Size 1, Gallenkamp) at a lower temperature (no more than 50°C) until it was ready to be ground. Plant components were then ground in a high-capacity grinding mill into coarse powders. The resultant powders were then kept in a dark, dry and cool atmosphere after being sealed in airtight containers. Dark-coloured flasks containing 538 g of processed dried plant material were sealed with 5.4 L of water. The infusions were filtered 24 h later. The procedure was repeated after 48 h. Using a rotary evaporator set at 40°C, the supernatants were vacuum-dried. Once the plant materials were determined to be depleted, the extraction process was considered complete. The plant material was then submerged in 1 L of deionised water and kept in a sealed container for 7 days, with occasional shaking and stirring. A fresh cotton bed filter was utilised for extraction. To create a sticky crude extract, the filtrates were dried at 40°C ± 2°C. After extraction, the materials were labelled appropriately and stored at 4°C in sterile sample containers [18].

### 2.2. GC-MS Analysis of Aqueous Extract From *Annona reticulata* L. Leaves

Shimadzu GC-MS-TQ8040 was utilised to perform GC-MS analysis. Helium (carrier gas) allowed for the successful separation and detection of the analytes. In accordance with GC parameters, the column oven's temperature was 50°C, ramped up to 200°C and finally reached 300°C. The hold times were 1, 2 and 7 min, respectively. The injection was conducted at 250°C, flow control mode set to pressure at 53.5 kPa and total flow of 11 mL/min. Under MS circumstances, temperatures of the ion source and interface were 230°C and 250°C. This configuration provides excellent sensitivity and specificity for identifying and quantifying volatile and semivolatile chemicals in complicated plant extracts by comparing them to library compounds [19].

### 2.3. In Vivo Studies

#### 2.3.1. Experimental Animals

Wister albino rats (120–230 g, male) and Swiss albino mice (25–30 g, male) were used in this study. The mice were gathered from the department's animal house. *Annona reticulata* L. leaf extract aqueous form was studied pharmacologically in the Jahangirnagar University Pharmacology Laboratory. In polypropylene cages with a suitable dark–light cycle, relative humidity (RH 55% ± 5%) and a temperature of 25°C ± 2°C, the animals were housed under standard laboratory conditions. The rats and mice were given ICDDR,B pelletised mouse feed and had unrestricted access to water. The instructions issued by Jahangirnagar University's animal ethics committee were followed in every action involving the care of animals.

#### 2.3.2. In Vivo Acute Toxicity Study

In order to ascertain the plant extract's safety profile for human consumption, an acute toxicity study was conducted. The work was conducted in accordance with the guidelines provided by Harizal et al. The OECD published 423 guidelines in 2001 for acute toxicity studies conducted in vivo. A total of three sets of five albino mice were gathered. The animals were kept famished through the night with unlimited water prior to the administration of the plant extract. Then, using an intragastric tube, the experimental animals were given *Annona reticulata* L. aqueous leaf extract at gradually higher concentrations, such as 1, 2 and 4 g/kg of body weight, in order to determine the optimum dosage. Following that, every animal was carefully monitored for any unusual behaviours that would point to any form of toxicity. Each animal was assessed separately for 14 days after taking medication, with particular attention paid to the first 4 h and then every day thereafter [20].

### 2.4. Evaluation of In Vivo Analgesic Activity

#### 2.4.1. Acetic Acid–Induced Abdominal Writhing in Mice

The approach described by Mondal et al. was utilised to investigate the analgesic effect of aqueous extract of *Annona reticulata* L. Firstly, five mice each were split up into four groups. Put a tail number on each mouse once they have been weighed. The mice in Groups I and II were designated as the control groups. They were given distilled water and diclofenac sodium (100 mg/kg), orally, respectively (as per body weight). The mice in Test Groups III and IV were administered 200 and 400 mg/kg of *Annona reticulata* L. aqueous leaf extract via the oral route. A 0.7% acetic acid injection was administered intraperitoneally to each animal 45 min after the dose was administered. The amount of time each mouse spent writhing over the course of 5 min was noted 15 min after the intake of acetic acid. Not all of the mice's writhing occurred at once. It would sometimes begin to writhe but cease before it could fully finish. These incomplete writhings were regarded as half writhings, and two of these half writhings equalled one full writhing. The overall writhing of two test groups was then contrasted with both the positive and negative controls, and using an established formula, the percentage of pain inhibition was determined [21, 22].

#### 2.4.2. Hot Plate Test in Mice

The hot plate method was utilised to investigate the analgesic properties of aqueous extract derived from *Annona reticulata* L. leaves, as documented by Mondal et al. First, four sets of five mice each were collected. Then, each mouse was weighed and given a tail number. Group I and Group II administered oral doses of distilled water (which was the negative control group) and 100 mg/kg of tramadol (standard/positive control), respectively, based on the body weight of each mouse. Mice in Groups III and IV were administered 200 and 400 mg/kg doses of aqueous leaf fraction. By placing each mouse on a hot plate that was kept at 55°C ± 2°C, pain was inflicted upon them using this manner. To reduce skin damage, the cut-off duration was set at 20 s [23]. Each animal's initial response time was measured. Pain response reaction times for each group were measured 30, 60, 90 and 120 min after the intake of doses, and the percentage analgesic activity was computed by utilising an established formula [22, 24].

#### 2.4.3. Formalin-Induced Paw Licking in Mice

The formalin-induced licking test was performed using a slightly modified version of the protocol outlined in Lucarini et al. Initially, four sets of five mice each were gathered. Following that, each mouse received a tail number and was weighed. The control groups were Groups I and II, where each mouse was given an oral dosage of distilled water and diclofenac sodium (100 mg/kg) based on body weight, respectively. For each mouse in Groups III and IV, the doses of aqueous extract were 200 and 400 mg/kg. Each mouse in four groups got 20 *μ*L of 2.7% formalin subplantarly injected into the left hind paw 30 min after the dose was administered. Two distinct stages were used to record how long each mouse bit and licked the injected paw. The first 5 min of the acute phase was characterised by behaviours including biting, licking, moving and lifting the injected paw. It immediately displayed the animal's reaction to the injection of formalin. More tonic behaviours, such as flinching and gripping the injected paw, were observed during the later chronic phase, which lasted between 25 and 30 min, and showed the mice's reaction to sustained pain stimuli, and the standard formula was utilised to compute the percentage inhibition of licking time [25, 26].

### 2.5. Assessment of In Vivo Anti-Inflammatory Activity

#### 2.5.1. Xylene-Induced Ear Edema in Mice

For assessing acute anti-inflammatory efficacy, the xylene-induced ear edema method in mice was used. After being gathered, mice were divided into four treatment groups, each with five animals: distilled water, 20 mL/kg; diclofenac sodium, 100 mg/kg; and two doses of aqueous extract (200 and 400 mg/kg). These treatments were given orally. One hour after oral treatment, xylene (20 *μ*L) was applied to the right ear's both surfaces in order to cause edema. The control ear was the left ear. Thirty minutes later, mice were killed by cervical dislocation. A cork borer (8 mm diameter) was used to remove mice's both ears, which were then weighed. The edema weight differential between the animal's right and left ears was computed, and utilising the following formula, the proportion of suppression of inflammation relative to the control group was calculated [27, 28]:

 $$\begin{array}{c} \%\text{Inhibition}=\left[\left(\text{WC}-\text{WT}\right)÷\text{WC}\right]\times 100. \end{array}$$

Here, WC = weight of edema (control) and WT = weight of edema (test).

#### 2.5.2. Cotton Pellet–Induced Granuloma in Rats

The chronic anti-inflammatory impact was evaluated using the cotton pellet–induced granuloma test. Five rats each were randomly assigned to one of four treatment groups. Two sterile cotton pellets, each weighing 20.00 ± 1.00 mg, were surgically inserted into each rat's groin area on the first day following anaesthesia (by intraperitoneal administration of ketamine hydrochloride) and shaving. The rats were given distilled water, diclofenac (10 mg/kg) and two doses of aqueous extract (200 and 400 mg/kg), intragastrially starting the day after surgery. Following a 7-day period, the rats were killed via cervical dislocation, and the granuloma-covered pellets were examined closely. Wet weights of the pellets were taken following removal of surrounding tissues. Once again, the weights of pellets were measured to ascertain their dry weight after being dried overnight at 60°C. The determination of weight variations between the test and control groups' wet and dry pellets was carried out, and the proportion of suppression of inflammation was computed utilising the following formula [27–29]:

 $$\begin{array}{c} \%\text{Inhibition}=\left[\left(\text{WC}-\text{WT}\right)÷\text{WC}\right]\times 100. \end{array}$$

Here, WC = weight of pellet (control) and WT = weight of pellet (test).

### 2.6. Evaluation of In Vivo Wound Healing Activity (Burn Wound Model)

#### 2.6.1. Creation of Burn Wound on Rats

Using ketamine, the rats were anaesthetised. The rats' backs were shaved to a 2 × 2 cm dimension and then rinsed with alcohol. Following anaesthesia, the process of creating burn wounds came next. A circular iron plate having an area of 300 mm^2^ was immersed in boiling water at 100°C for 5 min. Then, rats' backs were burned by applying the plate to them (30 s) [30].

#### 2.6.2. Treatment of Burn on Rats

A total of 20 male albino rats were randomly assigned to four groups, and each group was given four distinct treatments. The extract was used to create two different kinds of ointment compositions: 5% (*w*/*w*) and 10% (*w*/*w*), in which 100 g of basic ointment base was mixed with 5 or 10 g of test extract. The ointment base in this experiment was Vaseline petroleum jelly [31]. The various forms of treatment include

Group I: Rats with burns on their skin were given pure Vaseline petroleum jelly.

Group II: Skin-burned rats were given 1% silver sulfadiazine ointment.

Group III: 5% aqueous extract ointment was applied to rats having burns on their skin.

Group IV: Skin-burned rats were given 10% aqueous extract ointment.

Clean cotton buds were used to apply pure Vaseline petroleum jelly, *Annona reticulata* L. leaf extract ointments and silver sulfadiazine topically. For 21 days, the therapy was administered twice a day, with a 12-h break in between sessions.

#### 2.6.3. Assessment of Burn Wound

The parameters listed below were examined [31]:

1. Epithelialisation period: The epithelialisation period was determined by counting the number of days required for the wound's dead tissue remains to disappear without leaving any exposed wounds.
2. Wound contraction: The calculation of wound contraction was based on the percentage decrease in the wound area. The diameter was measured every other day (utilising calliper-based polygon approximation) in order to planimetrically track the progressive changes in the wound area.

On alternate days, the wound area was measured, and the epithelialisation period was also noted.

Statistical analysis was conducted using SPSS Version 27 for Windows and ANOVA. All findings were demonstrated as mean ± SEM, and *p* < 0.05 was deemed significant.

## 3. Result and Discussion

### 3.1. GC-MS Analysis Report of Aqueous Leaf Fraction From *Annona reticulata* L.

**Figure 1:**
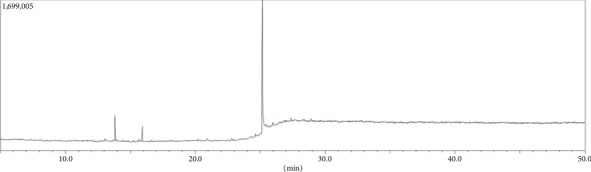
Total ionic chromatogram of *Annona reticulata* L. crude aqueous fraction.

A total of 78 components were identified in the crude aqueous leaf fraction of *Annona reticulata* L., according to the library search report (Figure 1). Among these substances, propenamide possesses potent analgesic potential [32], thiophene derivative and 13-docosenamide (z)- were found to have potent anti-inflammatory properties [33, 34], and cysteine helps wounds and burns recover [35].

### 3.2. In Vivo Studies

#### 3.2.1. Assessment of Acute Toxicity

The experimental animals were given dosages of *Annona reticulata* L.'s aqueous leaf fraction at 1, 2 and 4 g/kg body weight. Following a total of 14 days of observation, no signs of death, illness or aberrant behavioural changes were identified, suggesting that the extract was well tolerated. When giving extracts from *Annona reticulata* L., Shivanna et al. confirmed that there was no sign of immediate or delayed toxicity in experimental animals. As a result, giving the extract to test animals can be considered safe [36].

### 3.3. In Vivo Evaluation of Analgesic Activity

#### 3.3.1. Acetic Acid–Induced Writhing Test

In the acetic acid–induced writhing test, the negative control group showed 18.0 ± 1.08 writhings. After receiving diclofenac sodium treatment, the positive control/standard group demonstrated a noteworthy reduction in writhing to 6.25 ± 0.65, indicating a 65.28% (*p* < 0.001) inhibition. The 400 mg/kg dose of aqueous fraction from *Annona reticulata* L. leaves showed the strongest and statistically significant inhibition of 75% (*p* < 0.001) with a number of writhings of 4.50 ± 0.65, which is even greater than the standard group. Additionally, a 200 mg/kg dosage of aqueous leaf fraction demonstrated a significant analgesic effect by lowering writhing to 6.50 ± 0.65 with a 63.89% (*p* < 0.001) suppression (Table 1). Dose-dependent analgesic effect was seen in treatments using extract from *Annona reticulata* L. at different dosages.

**Figure 2:**
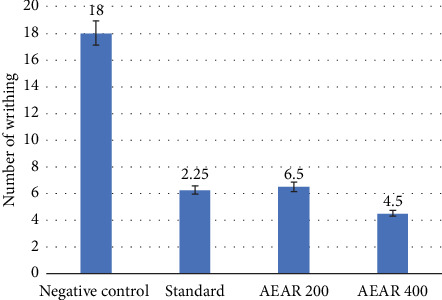
Effect of negative control, standard and AEAR in the acetic acid–induced writhing test.

Any drug's peripherally acting analgesic effects can be evaluated with the acetic acid–induced writhing test. Prostaglandin synthesis causes writhing via sensitising peripheral pain receptors [37]. The outcomes demonstrated that aqueous leaf fraction of *Annona reticulata* L. at 200 mg/kg and 400 mg/kg notably lowered pain response (Figure 2). One possible explanation for the analgesic action can be the inhibition of prostaglandin production.

### 3.4. Hot Plate Method

At various time intervals, the conventional tramadol treatment group, as well as two extract doses of aqueous leaf fraction (200 and 400 mg/kg) of *Annona reticulata* L., demonstrated a rise in latency period, compared to the negative control group. With inhibitions of 61.54% (*p* < 0.01), 78.57% (*p* < 0.001), 73.33% (*p* < 0.001) and 50% (*p* < 0.01) at 30, 60, 90 and 120 min, in due order, tramadol (standard) demonstrated considerable and long-lasting analgesic effect. Groups treated with the aqueous fraction of *Annona reticulata* L. leaves exhibited varying degrees of pain inhibition. The study found that the aqueous leaf extract, at a dose of 400 mg/kg, exhibited the maximum level of activity. At 30, 60, 90 and 120 min, the percentage analgesic activity was 53.85% (*p* < 0.05), 64.28% (*p* < 0.01), 80% (*p* < 0.001) and 56.25% (*p* < 0.001). It should be mentioned that, although aqueous extract initially performed worse than conventional tramadol, at 90 and 120 min, it outperformed the standard, indicating that its activity was longer lasting than tramadol. At a 200 mg/kg dose, aqueous leaf fraction exhibited some degree of analgesic efficacy with inhibition of pain values of 38.46%, 50% (*p* < 0.01), 60% (*p* < 0.01) and 37.5% (*p* < 0.05) at 30, 60, 90 and 120 min (Table 2). These outcomes demonstrated that test extract therapy had dose-dependent analgesic potential, with higher dosages producing larger increases in the latency period of pain.

**Figure 3:**
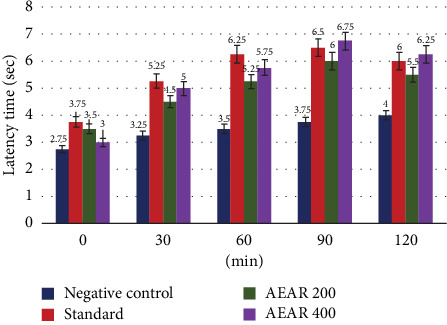
Effect of negative control, standard and AEAR in the hot plate test.

The hot plate method is a widely used tool for examining an animal's neuronal pain response. According to Vongtau et al. and Urquhart, centrally acting analgesics can lengthen the latency duration of the pain response in this test by acting at the spinal cord level [38, 39]. With percentages of pain inhibition of 60% and 80%, the extract at 200 and 400 mg/kg doses demonstrated the most notable effects at 90 min into the research (Figure 3). The outcomes obtained from the investigation demonstrated that *Annona reticulata* L. leaf aqueous fraction contained the capacity to reduce pain.

### 3.5. Formalin-Induced Paw Licking Test

**Figure 4:**
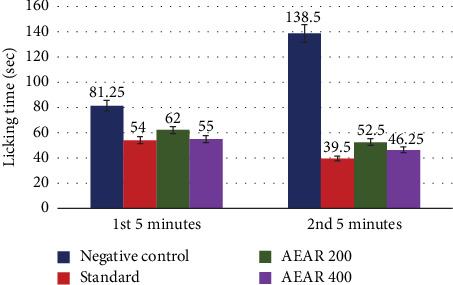
Impact of negative control, standard and AEAR in the formalin-induced paw licking test (1^st^ and 2^nd^ 5 min).

In the formalin-induced licking test, the negative control group's mean licking time was 81.25 ± 0.85 during the first 5 min and 138.50 ± 2.39 over the second 5 min. At the acute phase, the standard group that received diclofenac sodium showed a significant decrease in licking time, with a mean of 54.00 ± 0.91 (33.54% inhibition, *p* < 0.001), and at the chronic phase, with a mean of 39.50 ± 1.32 (71.48% inhibition, *p* < 0.001). In the case of a 400 mg/kg dosage of aqueous fraction, the licking time dramatically lowered to 55.00 ± 1.47 and 46.25 ± 1.80, which led to an acute phase pain response inhibition of 32.31% (*p* < 0.001) and a chronic phase pain response inhibition of 66.61% (*p* < 0.001), in due order. Furthermore, 200 mg/kg of aqueous extract decreased licking time to 62.00 ± 1.08 and 52.50 ± 1.44, while also inhibiting pain by 23.69% and 62.09% (Table 3). These results showed that the extract from *Annona reticulata* L. had analgesic effects that were dose-dependent (Figure 4).

Early and late phases correspond to the neurogenic and inflammatory phases, in this investigation [40]. The excitability of C-fibre nociceptors causes the neurogenic phase. The second phase is linked to central nociceptive mechanisms, including dorsal horn neuron activation that increases pain sensitivity [41]. The plant extract in the current investigation had a noteworthy impact throughout both the acute and chronic phases.

According to GC-MS analysis, the aqueous extract contained propanamide. Reports indicate that the chemical has potent analgesic effects [32]. Such analgesics work by directly inhibiting nociceptive activity, which travels up from the spinal cord's dorsal horn to brain circuitry linked to pain [42, 43]. Numerous investigators have assessed the analgesic efficacy of various extracts from *Annona reticulata* L. [44–46]. Furthermore, the results found in this study align with those of other studies, confirming aqueous leaf fraction from *Annona reticulata* L.'s analgesic potential.

### 3.6. Evaluation of In Vivo Anti-Inflammatory Potential

#### 3.6.1. Xylene-Induced Ear Edema Test

**Figure 5:**
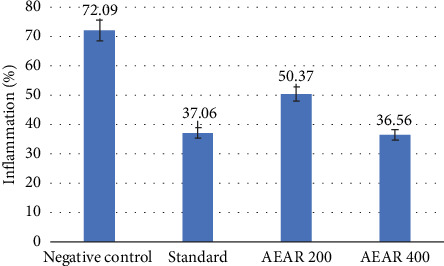
Effect of negative control, standard and AEAR in the xylene-induced ear edema test.

By comparing the edema weights of the animal's right (inflammatory) and left (control) ears, the xylene-induced ear edema test calculates the proportion of inflammation. The negative control group displayed a percentage of inflammation of 72.09 ± 1.68, whereas the standard group that took diclofenac sodium showed a percentage of 37.06 ± 2.52 (*p* < 0.001) with 48.59% inhibition of inflammation. Furthermore, an aqueous extract dose of 400 mg/kg demonstrated a positive outcome in this study, with an inflammatory value of 36.56% ± 2.42% (49.28% inhibition, *p* < 0.001). Additionally, even at a dosage of 200 mg/kg, the fraction had positive outcomes. But with the aqueous extract at 200 mg/kg, the proportion of inflammation was 50.37 ± 4.10 (30.13% inhibition, *p* < 0.001) (Table 4). Thus, it can be concluded that there was a definite dose-dependent anti-inflammatory activity of the extract (Figure 5).

Xylene-induced ear edema is a broadly utilised technique to assess the anti-inflammatory properties of natural products. As a phlogistic agent, xylene increases vascular permeability and causes the production of edema, which is a hallmark of acute inflammation. Xylene can quickly create acute inflammation in mice's ears by causing congestion and edema [47, 48]. The present investigation found that the test extract significantly reduced inflammation.

### 3.7. Cotton Pellet–Induced Granuloma Test

**Figure 6:**
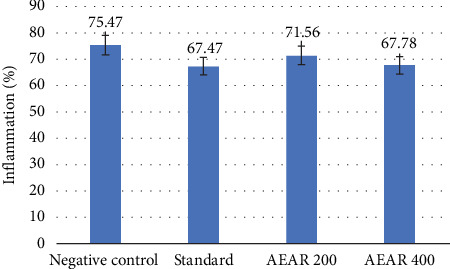
Effect of negative control, standard and AEAR in the cotton pellet–induced granuloma test.

A comparison of the weights of moist or dry pellets in the test and control groups was conducted to determine the proportion of inflammation. Aqueous fraction (400 mg/kg) notably lowered inflammation, with a percentage of inflammation of 67.78 ± 0.82 (10.2% inhibition, *p* < 0.001), compared to 75.47 ± 0.73 in the negative control group and 67.47 ± 1.34 (*p* < 0.001) in the standard group, which administered diclofenac sodium with 10.6% suppression of inflammation. Additionally, it was demonstrated that the extract was effective against inflammation even at a dosage of 200 mg/kg. When 200 mg/kg of aqueous extract was administered, the percentage of inflammation was 71.56 ± 0.69 (*p* < 0.05), and the suppression of inflammation was 5.2% (Table 5). Therefore, the extract indicated dose-dependent anti-inflammatory efficacy (Figure 6).

For assessing how successfully drugs combat the proliferative stage of inflammation, the cotton pellet–induced granuloma test is the best method [49]. Granuloma develops when a cotton pellet is placed subcutaneously in a rodent [50]. The investigation's results indicated that the fraction possessed potent anti-inflammatory activities.

Thiophene derivative and 13-docosenamide (z)- were found in the aqueous extract, according to GC-MS analytical findings. Each of these substances possesses potent anti-inflammatory properties [33, 34]. Anti-inflammatory agents work by blocking the cyclooxygenase (COX), an enzyme that produces prostaglandins [51]. A number of studies have evaluated the anti-inflammatory properties of several extracts from *Annona reticulata* L. [52–54]. The findings of our study resemble the outcomes of other researchers.

### 3.8. Assessment of In Vivo Wound Healing Activity (Burn Wound Model)

**Figure 7:**
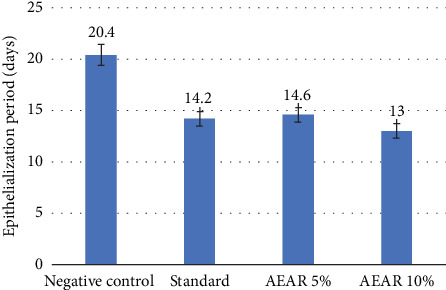
Effect of negative control, standard and AEAR on the epithelialisation period in the wound healing test.

For assessing healing properties, the wound's surface area was meticulously measured. To evaluate wound healing activity, the wound surface area was carefully measured and monitored on a regular basis.  Table 6 demonstrates that the negative control group exhibited the slowest rate of wound healing activity; the surface area of the wound was 300 mm^2^ on Day 0 and 2.4 ± 1.1 mm^2^ (99.2% of the wound contraction) on Day 21, with an epithelialisation period of 20.40 ± 0.40 days, respectively. Wounds in the standard group treated with silver sulfadiazine contracted faster; on Day 0, their surface area was 300 mm^2^, and on Day 12, it was 18 ± 2.2 mm^2^. On Days 3, 6, 9 and 12, the group's wound contraction percentage was 43.47%, 63.13%, 80.47% and 94%, respectively. The standard group also demonstrated a much faster epithelialisation time (14.20 ± 0.38 days; *p* < 0.001) than the negative control group. Good wound healing activity was also shown with 10% AEAR ointment; the wound surface area was 300 mm^2^ on Day 0 and 10.8 ± 1.9 mm^2^ on Day 12. On Days 3, 6, 9 and 12, the group's wound contraction percentage was 48.07%, 66.27%, 86.2% and 96.4%. Furthermore, the 10% AEAR ointment group epithelialised at average 13.00 ± 0.32 days (*p* < 0.001) faster than the conventional group. On Day 0 and Day 12, burn wound's surface area measurement was 300 mm^2^ and 14.8 ± 1.9 mm^2^, respectively, demonstrating the positive effects of the 5% AEAR ointment. For this group, the proportion of wound contraction was 43.27% on Day 3, 62.6% on Day 6, 83% on Day 9 and 95.07% on Day 12. The 5% AEAR ointment group demonstrated epithelialisation duration of 14.60 ± 0.25 days (*p* < 0.001), which was close to the value obtained from the standard group (Tables 6 and 7). Thus, a dose-dependent wound healing activity was shown by the extract in this study (Figure 7).

GC-MS analytical data revealed that compounds with wound healing activities were present in the aqueous extract, such as cysteine, which is highly beneficial for wound and burn healing [35]. By enhancing the body's capacity for angiogenesis, scavenging damaging free radicals and oxidative stress and promoting the migration and proliferation of fibroblasts (the cells that produce collagen and new tissue), cysteine aids in the healing of wounds. Additionally, it improves manganese superoxide dismutase activity and guards against ferroptosis (a process that can harm recovering tissues) [55]. In addition, the fact that the burn wounds were exposed to the outside world and that no bandage was used on them for the duration of the study is crucial. However, no microbial growth was seen in our investigation, which further supports the extract's potent antimicrobial potential. Nonetheless, to ensure the antimicrobial properties in open wounds, further testing is required in other wound models. As evidenced by progressive wound contraction and wound area reduction, the burn wounds demonstrated gradual proliferation and remodelling over the course of the 21-day observation period. After a few days, the first inflammatory phase ended, and the proliferation phase began, during which time visible granulation tissue most likely developed and promoted to close the wound. The wounds gradually strengthened and remodelled throughout time, as evidenced by greater tissue continuity and smoother wound edges by Day 21. These macroscopic alterations indicated that the skin surface was functionally restored as a result of the restoration processes moving forward successfully. Proliferation and remodelling are slow processes that eventually restore tissue integrity, and this pattern observed in our study was consistent with the normal time course of burn wound healing. Furthermore, Mazumdar et al. have assessed and validated the wound healing properties of *Annona reticulata* L.'s ethanolic leaf fraction [56]. Nevertheless, this study was the first to assess the aqueous leaf fraction from *Annona reticulata* L.

Our current study utilised a small sample size (*n* = 5), which would have limited the conclusions' statistical power. To get more precise and reliable outcomes, the sample size might be increased. Additionally, histopathological examinations could be included to evaluate the progression of wound healing more accurately. In addition, follow-up periods can be extended to determine whether any delayed adverse effects occurred. Furthermore, the study only utilised rodent models; hence, it was unable to predict the outcomes in other animal models. For this reason, similar investigations should be carried out in other animal models to determine the activities by observing how the test extract performs in those animals. Alongside *p* values, effect sizes and confidence intervals could also be included to enhance statistical reporting.

## 4. Conclusion

In mice, aqueous leaf extract of *Annona reticulata* L. demonstrated good pain-inhibiting properties in three in vivo analgesic assays. Xylene-induced ear edema and cotton pellet–induced granuloma tests demonstrated the test fraction's noteworthy anti-inflammatory potential. To assess the aqueous leaf extract's significant wound healing properties, a burn wound model was utilised, which indicated the aqueous leaf extract's potent wound healing properties. The plant constituents that aided in the achievement of these activities were also identified by GC-MS analysis. Despite the apparent promise of analgesic, anti-inflammatory and wound healing potentials in animal models, more thorough research is needed to fully comprehend the exact mechanism of action behind these activities.

## Figures and Tables

**Table 1:** Effect of negative control, standard and AEAR in the acetic acid–induced writhing test.

**Group**	**Number of writhing (** **m** **e** **a** **n** ± **SEM)**	**% inhibition of pain**
Negative control	18.0 ± 1.08	_
Standard	6.25 ± 0.65^∗∗∗^	65.28%
AEAR 200	6.50 ± 0.65^∗∗∗^	63.89%
AEAR 400	4.50 ± 0.65^∗∗∗^	75%

*Note:* The standard is diclofenac sodium.

Abbreviation: AEAR, aqueous extract of *Annona reticulata* L. leaves.

⁣^∗^*p* < 0.05 (significant), ⁣^∗∗^*p* < 0.01 (highly significant) and ⁣^∗∗∗^*p* < 0.001 (very highly significant) against control.

**Table 2:** Effect of negative control, standard and AEAR in the hot plate test.

**Group**	**Latency time in hot plate (** **m** **e** **a** **n** ± **SEM)**				
	**0 min**	**+30 min**	**+60 min**	**+90 min**	**+120 min**
Negative control	2.75 ± 0.48	3.25 ± 0.25	3.50 ± 0.29	3.75 ± 0.48	4.00 ± 0.41
Standard	3.75 ± 0.25 (36.36%)	5.25 ± 0.48^∗∗^ (61.54%)	6.25 ± 0.25^∗∗∗^ (78.57%)	6.50 ± 0.29^∗∗∗^ (73.33%)	6.00 ± 0.41^∗∗^ (50%)
AEAR 200	3.50 ± 0.29 (27.27%)	4.50 ± 0.29 (38.46%)	5.25 ± 0.48^∗∗^ (50%)	6.00 ± 0.41^∗∗^ (60%)	5.50 ± 0.29^∗^ (37.5%)
AEAR 400	3.00 ± 0.41 (9.09%)	5.00 ± 0.41^∗^ (53.85%)	5.75 ± 0.25^∗∗^ (64.28%)	6.75 ± 0.25^∗∗∗^ (80%)	6.25 ± 0.25^∗∗∗^ (56.25%)

*Note:* Parentheses indicate the percentage of analgesic activity (%A) in relation to the control group. The standard is tramadol hydrochloride.

Abbreviation: AEAR, aqueous extract of *Annona reticulata* L.

⁣^∗^*p* < 0.05 (significant), ⁣^∗∗^*p* < 0.01 (highly significant) and ⁣^∗∗∗^*p* < 0.001 (very highly significant) against control.

**Table 3:** Effect of negative control, standard and AEAR in the formalin-induced paw licking test (1^st^ and 2^nd^ 5 min).

**Group**	**1 ** ^**st**^ ** 5 min (acute phase)**		**2 ** ^**nd**^ ** 5 min (chronic phase)**	
	**Licking time (sec) (** **m** **e** **a** **n** ± **SEM)**	**% inhibition of Algesia**	**Licking time (sec) (** **m** **e** **a** **n** ± **SEM)**	**% inhibition of Algesia**
Negative control	81.25 ± 0.85	_	138.50 ± 2.39	_
Standard	54.00 ± 0.91^∗∗∗^	33.54%	39.50 ± 1.32^∗∗∗^	71.48%
AEAR 200	62.00 ± 1.08^∗∗∗^	23.69%	52.50 ± 1.44^∗∗∗^	62.09%
AEAR 400	55.00 ± 1.47^∗∗∗^	32.31%	46.25 ± 1.80^∗∗∗^	66.61%

*Note:* The standard is diclofenac sodium.

Abbreviation: AEAR, aqueous extract of *Annona reticulata* L.

⁣^∗^*p* < 0.05 (significant), ⁣^∗∗^*p* < 0.01 (highly significant) and ⁣^∗∗∗^*p* < 0.001 (very highly significant) against control.

**Table 4:** Effect of negative control, standard and AEAR in the xylene-induced ear edema test.

**Group**	**% inflammation (** **m** **e** **a** **n** ± **SEM)**	**% inhibition of inflammation**
Negative control	72.09 ± 1.68	_
Standard	37.06 ± 2.52^∗∗∗^	48.59%
AEAR 200	50.37 ± 4.10^∗∗∗^	30.13%
AEAR 400	36.56 ± 2.42^∗∗∗^	49.28%

*Note:* The standard is diclofenac sodium.

Abbreviation: AEAR, aqueous extract of *Annona reticulata* L.

⁣^∗^*p* < 0.05 (significant), ⁣^∗∗^*p* < 0.01 (highly significant) and ⁣^∗∗∗^*p* < 0.001 (very highly significant) against control.

**Table 5:** Effect of negative control, standard and AEAR in the cotton pellet–induced granuloma test.

**Group**	**% inflammation (** **m** **e** **a** **n** ± **SEM)**	**% inhibition of inflammation**
Negative control	75.47 ± 0.73	_
Standard	67.47 ± 1.34^∗∗∗^	10.6%
AEAR 200	71.56 ± 0.69^∗^	5.2%
AEAR 400	67.78 ± 0.82^∗∗∗^	10.2%

*Note:* The standard is diclofenac sodium.

Abbreviation: AEAR, aqueous extract of *Annona reticulata* L.

⁣^∗^*p* < 0.05 (significant), ⁣^∗∗^*p* < 0.01 (highly significant) and ⁣^∗∗∗^*p* < 0.001 (very highly significant) against control.

**Table 6:** Effect of negative control, standard and AEAR on wound contraction in the burn wound model.

**Postwounding days**	**Wound surface area (** **m** **e** **a** **n** ± **SEM) in mm **^**2**^** and percentage of wound contraction**			
	**Negative control**	**Standard**	**5% AEAR**	**10% AEAR**
0	300	300	300	300
3	244.4 ± 2.8 18.53%	169.6 ± 2.1 43.47%	170.2 ± 3.7 43.27%	155.8 ± 1.3 48.07%
6	212 ± 4.1 29.33%	110.6 ± 2.2 63.13%	112.2 ± 4.9 62.6%	101.2 ± 4.2 66.27%
9	174.4± 2.8 41.87%	58.6 ± 2.4 80.47%	51 ± 1.6 83%	41.4 ± 2.4 86.2%
12	131.4 ± 2.9 56.2%	18 ± 2.2 94%	14.8 ± 1.9 95.07%	10.8 ± 1.9 96.4%
15	88.8 ± 1.9 70.4%	_	_	_
18	24.8 ± 1.9 91.73%	_	_	_
21	2.4 ± 1.1 99.2%	_	_	_

*Note:* The standard is silver sulfadiazine.

Abbreviation: AEAR, aqueous extract of *Annona reticulata* L.

**Table 7:** Effect of negative control, standard and AEAR on the epithelialisation period in the wound healing test.

**Group**	**Epithelialisation period (days) (mean ± SEM)**
Negative Control	20.40 ± 0.40
Standard	14.20 ± 0.38⁣^∗∗∗^
AEAR 5%	14.60 ± 0.25⁣^∗∗∗^
AEAR 10%	13.00 ± 0.32⁣^∗∗∗^

*Note:* The standard is silver sulfadiazine, and the negative control is Vaseline petroleum jelly.

Abbreviation: AEAR, aqueous extract of *Annona reticulata* L.

⁣^∗^*p* < 0.05 (significant), ⁣^∗∗^*p* < 0.01 (highly significant) and ⁣^∗∗∗^*p* < 0.001 (very highly significant) against control.

## Data Availability

All data and materials are contained and described within the manuscript.
